# Institutional Review Board Preparedness for Disaster Research: a Practical Approach

**DOI:** 10.1007/s40572-021-00311-x

**Published:** 2021-05-11

**Authors:** Joan P. Packenham, Richard Rosselli, Alice Fothergill, Julia Slutsman, Steve Ramsey, Janet E. Hall, Aubrey Miller

**Affiliations:** 1 Office of Human Research Compliance, Clinical Research Branch and Division of Intramural Research, National Institute of Environmental Health Sciences, 111 T.W. Alexander Dr., Mail Drop CRU-02, Research Triangle Park, Durham, NC 27709 USA; 2 Social & Scientific Systems, Inc., Silver Spring, MD USA; 3 College of Arts and Sciences, Department of Sociology, University of Vermont, Burlington, VT USA; 4 Office of Director, Office of Extramural Research, National Institutes of Health, Bethesda, MD USA; 5 Clinical Research Branch and Division of Intramural Research, National Institute of Environmental Health Sciences, Durham, NC USA; 6 Office of Director, National Institute of Environmental Health Sciences, Durham, NC USA

**Keywords:** Disaster research, Ethics, Vulnerability, Institutional Review Board (IRB), Preparedness, Protocol development

## Abstract

**Purpose of Review:**

Disasters are becoming more common and challenge national and global resiliency and response efforts. As a result, government agencies have increased interest in disaster research to understand their environmental impact and health-related consequences. With the research field greatly expanding, Institutional Review Boards (IRBs) are being asked to review research protocols aimed at assessing health risks, exposures, and outcomes from disaster survivors. Few IRBs have experience reviewing disaster research protocols. This article describes approaches for IRB preparedness in reviewing disaster research.

**Recent Findings:**

From a human research protections perspective, primary attention has focused on vulnerability of individuals and/or populations affected by a disaster who may serve as research participants [3, 4]. From our review of the current literature, there is a lack of best practices and/or guidance for IRBs in the review of disaster research protocols.

**Summary:**

The growth of the disaster research field has brought more attention to potential ethical concerns of disaster research studies. Disaster survivors, responders, and those that assist in cleanup and remedial efforts may be left with significant unmet needs and long-term physical and emotional challenges as a result of their experiences. It is important for IRBs and investigators to collaboratively address how best to protect the welfare of individuals and communities affected by a disaster. A new approach is needed to systematically consider the various factors relevant to an assessment of human research protection issues following disasters.

## Background

The increased impact of health emergencies and disaster events over the last several decades [1] has been accompanied by an increase in research designed to understand their environmental and health-related consequences [2]. Disaster research engages a diverse mix of scientific disciplines in studies ranging from post-disaster observational surveys to epidemiological assessments to interventional clinical trials. The importance of this research is clearly underscored today by one of the largest worldwide public health disasters in modern history, the ongoing response to the 2019–2021 coronavirus pandemic. In the midst of this pandemic, teams of scientists from many disciplines are collaborating across the globe to develop an understanding of COVID-19. Together, they are finding ways to reduce/control the spread by developing surveillance tools and diagnostic testing, developing therapies and vaccines through clinical interventions, and to understand public health and societal consequences via occupational health and safety practices and social, behavioral, and educational research and interventions.

The growth of the disaster-related health research field in response to these events has brought more attention to the potential ethical concerns of these studies. Particular attention has been focused on the vulnerability of individuals and/or populations affected by a disaster who may be asked to participate in research [3, 4]. Nowhere is this more apparent than during the pandemic when vulnerable populations such as the elderly, persons living in congregate housing, those with medical comorbidities, and racial/ethnic minorities are suffering disproportionate impact from COVID-19. Disaster survivors, responders, frontline workers, and those that assist in cleanup and remedial efforts may be left with significant unmet needs and long-term physical and emotional consequences as a result of their experiences. While such experiences do not necessarily confer uniform vulnerability among exposed groups [5–7], it is important for Institutional Review Boards (IRBs) to assess whether an investigator has considered and addressed how best to protect the welfare of individuals and communities affected by a disaster.

The protection of human subjects in the United States is codified within the Code of Federal Regulations (Revised Common Rule: 45 CFR 46, 2018, Food and Drug Administration: 21 CFR Parts 50, 56) which establishes IRBs as the reviewing entity to assure that appropriate safeguards exist to protect the rights and welfare of research subjects and sets out criteria for IRB approval of research (Revised Rule: 45 CFR 46.111). In carrying out these responsibilities, IRBs have established standard review criteria and tools that allow a proper assessment of the suitability of proposed research. Most IRBs utilize a checklist or multiple checklists with standard questions for reviewers to consider as they review a research protocol. Such checklists include sections on research design, subject selection, risks and benefits, confidentiality, remuneration, and the informed consent process. While such IRB review checklists are broadly applicable, disaster research may present unique issues that are not sufficiently addressed by standard review criteria. Therefore, a new approach is needed for IRBs and investigators to systematically consider the various factors relevant to an assessment of human research protection issues in disaster-related research. This approach is a key component of a comprehensive IRB preparedness program being developed by the NIEHS Disaster Research Response (DR2) Program Office of Human Research Compliance (OHRC) that will include practical tools, training modules, and best practices.

## Objectives

The goal of this paper is to further develop IRB preparedness through the introduction of best practices to enhance the ethical review of disaster research protocols. The Disaster Research Critical IRB Review Factors Model is a practical approach for IRBs to use in the review of disaster-related protocols. The NIEHS IRB disaster preparedness training program utilizes this model in combination with disaster research review tools and case studies. The NIEHS IRB preparedness training program is unique in that it brings together principal investigators (PIs) with expertise and/or interest in disaster research with IRB members and staff prior to disasters to build relationships and conduct training on special considerations in the development and review of disaster-related protocols. In 2015, the Office of Human Research Compliance (OHRC) at NIEHS formed the NIEHS Best Practices Working Group for Special IRB Considerations in the Review of Disaster Related Research. This national working group met in 2016 to discuss ethical concerns raised by disasters and ways to improve the IRB review of disaster research. Major thematic elements from these discussions were collected and published as recommendations of the working group [8]. While these recommendations provided general guidance to IRBs and investigators, there remained a need for a more specific guidance on implementing best practices to address the unique circumstances of reviewing disaster research protocols. Through careful consideration of each recommendation, the NIEHS OHRC identified several critical factors that are essential to conducting a thorough IRB review of disaster research protocols.

## Disaster Research Review Model

A key difference between routine public health or clinical research and research conducted in the wake of a disaster is the approach that a dynamic disaster situation requires. The conditions surrounding the aftermath of a disaster can change rapidly, depending on the extended consequences of the disaster (e.g., number of victims, rescue/recovery challenges, and health concerns). Therefore, review of disaster research requires a more critical look at normal review factors as well as additional review factors. To illustrate this concept, the Disaster Research Critical IRB Review Factors Model has been developed to aid in the review and approval of research.

**Fig. 1:**
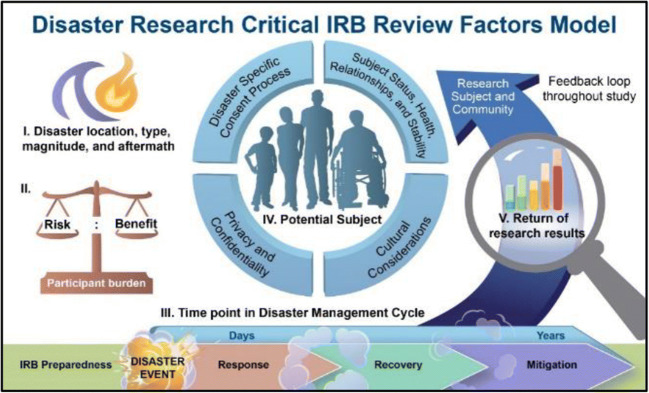
Disaster Research Critical IRB Review Factors Model

Figure 1 portrays five critical review factors relevant to the review of disaster research:

1. Disaster location, type, magnitude, and aftermath;
2. Risks/benefits to participants and study team;
3. Time point in the Disaster Management Cycle;
4. Status of potential subjects, and
5. Return of research results to participants and the affected community.

Some of these factors are unique to disaster research, while others overlap with criteria already used in IRB review. Each factor includes subfactors that are critical to an IRB’s thorough consideration of a proposed disaster research study. The NIEHS OHRC is developing and piloting a disaster research review checklist which is a companion to the model and is intended to be used in tandem with an IRB’s existing standard review checklist. The checklist aligns with the model’s factors, providing points to consider (i.e., special considerations) to assist reviewers in an assessment of the adequacy of human research protections in disasters. The balance of this paper will discuss each of the five factors included in the model and describe how each factor contributes toward the development of a new practical framework for IRB review of disaster research.

## Discussion

### The Five Model Factors

#### Disaster Location, Type, Magnitude, and Aftermath

Though there is no single, agreed upon definition of disaster either within or across disciplines, the United Nations [9] defines a disaster as “a serious disruption of the functioning of society, causing widespread human, material, or environmental losses which exceed the ability of affected society to cope using only its own resources.” Some disasters occur as a result of natural hazards and include events such as flooding, tornados, hurricanes, ice storms, volcanic eruptions, drought, and pandemics, while non-natural incidents include terrorist attacks, mass shootings, radiation releases, toxic chemical releases, and civil conflicts [10]. In either case, the impacts of the disaster are based largely on the geographic spread and the size of the population impacted.

IRBs and investigators need to be cognizant of the broader landscape and consider the status of the impacted community and the environment as a whole. Central to this consideration is the functional status of the community and the accessibility of basic needs. Researchers should consider whether the community has access to food, shelter, working utilities, and health care services and factor this into the review of the protocol submitted to the IRB. Some disasters may play out over long periods of time resulting in far-reaching impacts on critical community needs. For example, the disruptions caused by the ongoing COVID-19 pandemic have led to major socioeconomic changes including high unemployment, weakened supply chains, and increased poverty and food insecurity. Another example, Hurricane Maria in Puerto Rico, illustrates how long communities may go without basic needs, such as electricity, water, and food, challenging conventional wisdom about post-disaster timelines [11]. In addition, if research is being conducted with evacuees in a new location, such as a neighboring county or state, it is important to consider the environment outside the disaster area where research may need to be conducted. Large-scale displacement of residents may be a critical issue after disasters, and disaster survivors may have difficulty finding a stable location to settle once uprooted. After Hurricane Katrina, for example, evacuees often experienced multiple moves before obtaining stable housing [12]. Displacement is a difficult, stressful process [13]. Those who are displaced may stay in mass shelters, rental units, FEMA trailers, or with family and friends. Past research has found that utilization of mass shelters for disaster survivors depends on various factors which may affect their choices (e.g., health care access, medication needs, concern for pets or property, separation from family, and special medical needs), including their resources to stay elsewhere [14]. While being displaced should not preclude an individual from being a research participant, a proper assessment of the risks, benefits, and vulnerabilities of displaced individuals should be included in research plans. Displaced individuals living in mass shelters are commonly targeted [14–16] for disaster research recruitment efforts due to sampling convenience. Therefore, participant burden and/or research fatigue is of concern and should be considered by IRBs and investigators. Mass shelters also may be a challenging setting for protecting research participants due to the lack of privacy/confidentiality and potential security concerns.

IRBs were established to primarily protect individual research participants, and thus, even with community representation on the board, overall community considerations may be absent in review processes [17]. Disasters may weaken communities and their socioeconomic structures to the point that they may become susceptible to exploitation. The IRB can play a key role in protecting the disaster-impacted community through assuring that the proposed research is feasible (e.g., likely to achieve recruitment goals to yield meaningful results) and has established necessary relationships for successful community research engagement. A key feature of researcher preparedness is attaining a proper understanding of the affected community prior to the disaster. Many disaster-impacted communities have a long history of experience with disasters due to geography (e.g., Atlantic hurricane zone) or underlying susceptibility (e.g., earthquakes in a seismic zone). Disaster research teams conducting post-disaster studies in such regions should be familiar with these communities beforehand in order to understand the unique demographic and cultural features needed to inform the research and to build trust. Since it is not as clear whether the board will be familiar with all of the ramifications of each type of disaster, as much situational intelligence as possible should be obtained by investigators and/or consultants and made available to IRBs reviewing disaster research protocols. For example, it has been observed that there are vulnerable communities and populations as it relates to susceptibility to disease, recovery, and mortality from COVID-19. Researchers and IRBs need to have situational awareness of the cultural, economic, and health considerations of these populations to adequately perform and review research on these vulnerable populations.

Examples of pre-context considerations include long-term health disparities, preexisting health conditions, lack of personal control of exposure to disease (ex. lack of workplace safety considerations such as PPE, inadequate testing, and vaccinations), and lack of adequate health insurance, and accessibility to health care. Considerations of these issues should be factored into the design of the various components and their review of protocols to adequately define the current status of potential participants, analysis of risks and benefits, research burden on these populations, as well as including a plan for reporting back research findings to individuals and communities. NIEHS is currently piloting a preparedness tool (Pre- and Post-Disaster Researcher Engagement Assessment and Community Template or PD-REACT) that will help provide IRBs with context about the location, type, magnitude, and aftermath of the disaster. PD-REACT is a customizable tool intended to be completed by the research team and submitted to the IRB along with the research protocol and other supplementary review material and helps the IRB determine if the investigator has a firm grasp of the pre- and post-disaster community.

#### Risks/Benefits to Participants and Study Team

The assessment of risks and benefits in the review and approval of research is a requirement of IRB review. Disasters may create new daily norms, such as physical distancing and quarantine practices during the COVID-19 pandemic, for affected populations and potential research participants. This may make the determination of risk challenging for IRBs.

Navigating the ethical issues of disaster research requires additional considerations and sensitivity. Following approval of a protocol, IRBs should consider the appropriate frequency of continuing review, particularly for greater than minimal risk studies. In some cases, it may be appropriate for IRBs to require more than the typical annual continuing review. Situations during and after disasters are constantly shifting, and IRBs need to ensure that protection of participants and researchers is being maintained throughout the study period. Given the speed in which the disaster research environment may change, IRBs could consider setting up a near real-time review process whereby researchers in the field can quickly access the IRB office and the IRB chair to allow for consultation and facilitate development of approvable protocol amendments that adequately reflect needs in the disaster field. While real-time IRB responsiveness may only be necessary in challenging circumstances or cohorts, interaction between the PI and the IRB needs to be ongoing and not merely reserved to the time period prior to study initiation. Due to unpredictability in the disaster field, IRBs should also find creative ways to work with investigators to address ethical issues in a timely manner. During the review of the protocol, IRBs should provide clear guidance about timelines for PIs to report back and/or check in concerning study progress and potential issues. It is only in this way that important research can be accomplished, thus contributing to knowledge, and improving outcomes for future victims and their families while ensuring that risks to research participants are minimized [5••].

One issue that has been widely debated in the disaster research ethics literature is participant vulnerability. According to a review of existing ethical guidelines for post-disaster research, vulnerability is a core issue for researchers around the globe [18]. While various types of vulnerability do not preclude individuals from research participation, they do indicate the need for careful planning and ethical considerations [19]. Trauma-focused research often requires participants to recall potentially painful memories [20]. Although emotional distress is not unique to participants of disaster research, IRBs are often concerned that participants will be “retraumatized” when asked to recall traumatic events [5, 21]. The literature is equivocal with regard to whether such research exacerbates existing vulnerabilities. The disaster phase in which research is initiated likely plays a key role. Whereas some authors have shown that, in general, participation in most post-disaster research is not emotionally detrimental to disaster survivors [21], others demonstrate potential harms [4, 22, 23].

While there is not explicit language in current humans subjects protections regulations that categorizes disaster survivors as vulnerable subjects, the National Bioethics Advisory Committee [24] outlined situational circumstances (e.g., emergencies/disasters) that may contribute to cognitive vulnerability. It is reasonable to assume that some disaster survivors may have some mental sequelae or physical stress associated with the incident, especially in the immediate aftermath of the event. For example, results from the NIEHS GuLF Long-term Follow-up Study illustrate the magnitude of mental health issues within a disaster worker cohort following the Gulf oil spill , and all participants had the potential of benefiting from referrals to mental and physical health services. [22•]. Other environmental studies, as discussed in a review of quantitative studies, indicate that disasters have a clear impact on mental health, with the two most common adverse outcomes being depression and post-traumatic stress disorder [23]. The significant disruption in daily norms and interrupted access to behavioral health services during most disasters has led to increased concerns about global mental health [25]. IRBs and investigators should be aware of these disaster-related health outcomes as potential risks and consider them as well as the capacity of participants to give informed consent in these circumstances. As disaster situations are not business as usual, the disaster team may want to consider a multidisciplinary research team where team members who are trained in social behavioral sciences such as social workers or clinical psychologists are added to assist in the assessment of potential participants. The NBAC also describes social and economic vulnerability, which may particularly apply to disasters which can damage social networks and livelihoods leaving potential subjects susceptible to coercion and undue influence [24].

An assessment of the safety and welfare of the research participant must also take into account the type of proposed research. Affleck [26] argues that, “…separation of therapeutic and non-therapeutic research procedures is a fundamental principle of the risk assessment.” While therapeutic research may employ a traditional risk-benefit ratio, where higher risks may be acceptable as long as they are offset by direct benefits, non-therapeutic research is not intended to meet the participant’s health needs and may not confer direct benefit. Participants in non-therapeutic research may experience indirect benefits, but the risks associated with such studies should not be measured against such benefits. IRBs should be conscious of this distinction when considering disaster research, especially if the proposed research is to be conducted during the disaster or in the immediate aftermath of a disaster. For non-therapeutic research, minimization of risks should be prioritized, followed by an assessment of the intended scientific value compared to the potential risks.

Following disasters, many researchers may be interested in studying how communities have been affected, and it is possible that individuals could be approached by multiple researchers. A unique and commonly cited risk for participants of disaster research is enrollment in redundant studies which can overly burden or fatigue the community [5, 29]. This is a looming issue in the current pandemic where we have seen prolific growth in COVID-19-related studies potentially leading to research fatigue among prospective research participants [27]. Collogan et al. provide an example of how, under executive order of the governor of Oklahoma, the University of Oklahoma’s IRB became the central approving body for research of victims following the Oklahoma City bombing in 1995. As a result, overall burden to participants was minimized, knowledge gained maximized, and simultaneous study needs satisfied. Because of the possibility of redundant studies and increased participant burden following disasters, PIs, IRBs, institutional officials, and city and county governments should work collaboratively to develop strategies for coordinating with communities and other universities and health agencies to reduce the possibility of increasing the burden on participants.

Another example of a successful approach to reduce the risk of redundant studies and minimize participant burden was undertaken after Hurricane Harvey [28]. Researchers, media, state and local agencies, and nonprofit organizations throughout the region conducted environmental and biological sampling, community health assessments, and surveys/registries in a coordinated fashion through participation in pre-established academic-practice partnerships formed through the DR2 Program. The various stakeholders worked to improve coordination and research translation to the public. An additional example taking place during the COVID-19 pandemic is seen at the University of Washington (UW). Due to an overwhelming number of requests for patients (participants), biospecimens, access to medical records, etc., from university investigators and known and unknown investigators across the nation and world, the UW has worked to coordinate research interest groups to address research needs, reduce redundancy and participant burden, finding ways to make data sharing more available, and adding COVID-19-related questions to existing patient surveys and questionnaires. The success of these processes should serve as an example for the research community as a strategy to promote research collaboration across multiple institutions during or in the wake of disasters. IRBs must also consider potential risks to the researchers. The safety of the disaster and post-disaster fields are paramount as unprepared researchers or disaster research teams can place additional burdens on the responder community at a crucial time. A proper assessment of ongoing and post-disaster hazards coupled with research team training is essential to minimize researcher risks. In assessing researcher readiness, study teams should demonstrate to the IRB previous experience working with disaster victims and/or training in disaster research conduct, as well as knowledge about the community of interest. Furthermore, protocols should detail plans for the training and support of staff that may be exposed to challenging situations [5••]. Research staff may be approaching prospective research participants at one of the most difficult times in their lives and must be trained on how to defuse and triage crises should they occur. Aside from pre-deployment training in psychological first aid, research teams can improve their preparedness by assuring that they have access to diverse disciplines in the field, such as medical and behavioral health professionals. In addition, appropriate and meaningful systems of referrals for those in need must be considered and built into the research response. An additional consideration of risks/benefits to pariticipants is the determination of minimal risk or greater than minimal risk studies. This is an important IRB decision which may have significant consequences on the approval process. Minimal risk studies, for example, are often considered for expedited review and may not have to undergo continuing review. A reassessment of risks and benefits within disaster research, however, may be warranted as the IRB standard used to measure risk against those ordinarily encountered in daily lives may be fundamentally altered during and after disasters.

#### Time Point in the Disaster Management Cycle

**Fig. 2:**
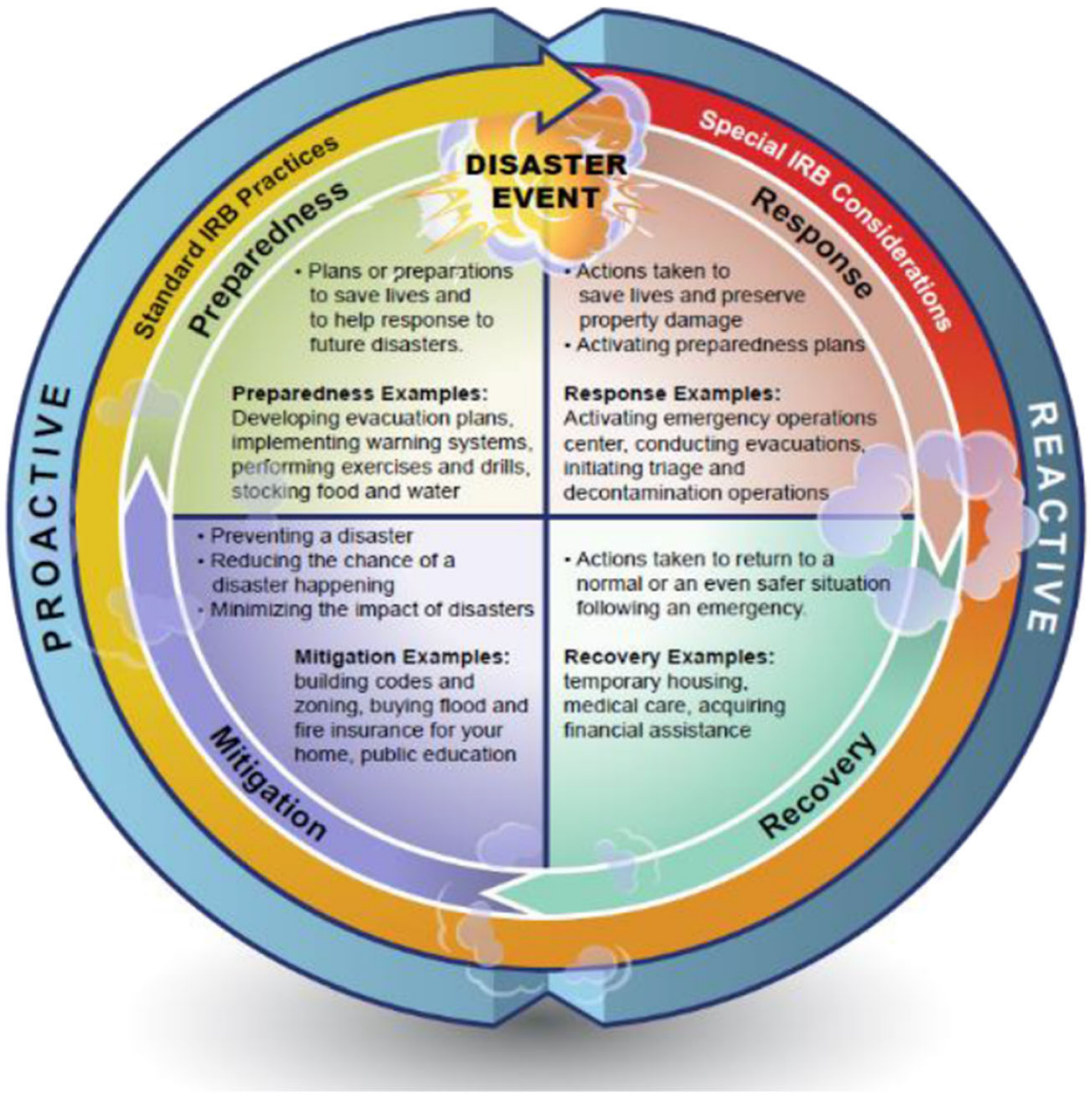
Model depicting the disaster management phases in a cycle and examples of the types of IRB-related activities conducted during each one

In 1979, the National Governor’s Association published a report that described the four phases of emergency management, which has led to the widespread use of “mitigation, preparation, response, and recovery” to help describe comprehensive emergency management of disasters. These phases are often displayed as a cycle illustrating the ongoing process by which communities plan for and reduce the impact of disasters. Though four phases are named in the cycle, there is no clear distinction between one phase ending and another beginning. A community will always occupy at least one phase of the cycle, but it is also possible to be in more than one phase at any given time (Fig. 2). Disaster research planning and operations can be conducted in any of the four phases of the cycle. A review board should expect information within a protocol for how the research team has planned its work so as not to conflict with emergency responders or ongoing recovery if research is to be conducted during the response or recovery phases.

During the preparedness and mitigation phases, most communities would be considered to be in their “normal” state. Disaster research studies initiated during this “pre-impact” stage would likely be carried out without special IRB considerations. There may be a need to collect data during the preparedness phase and throughout the response and recovery phase to better understand the impact of the disaster on the community of interest. For instance, if a hurricane was developing over open water and was expected to make landfall in a matter of days, one may wish to take repeat measures from the population of interest before, during, and after the disaster. The response phase is generally the most concerning from an ethical standpoint and as such, special IRB considerations should be brought into the review process. If research activities are occurring in concert with response activities, review boards should consider whether the level of coordination between emergency managers and researchers is adequate. For instance, review boards may want to ensure that researchers are prepared to integrate into the ongoing response which may be using a standardized hierarchical structure like the Incident Command System (ICS) that enables a cooperative response by multiple agencies and organizations and that research activities do not compromise the decision-making authority of local incident management leaders or interfere with emergency response operations. It is also important to safeguard against unintended coercion so that participants are not confusing the research staff with the responder community. Efforts should be made to make a clear distinction between the researchers and disaster workers and the myriad of other personnel that may descend upon the ongoing or post-disaster field through badge identification, attire, or other clearly identifying method.

At any time in the disaster management cycle, concerns may arise regarding resource allocation. Because research activities and emergency response activities might require different procedures to accomplish their respective ends, they can make competing demands on time, personnel, and other social and economic resources. Consideration should be given to making sure that resources are being allocated to the most essential needs of the individual or community. For example, at the onset of the COVID-19 pandemic, there was limited supply of personal protective equipment (PPE) across the nation, such that the needs of essential medical personnel outweighed any researcher demands. An assessment should be undertaken to review the type and amount of research information being collected to assure that it is appropriate given the status of the situation. In some cases, it may be acceptable to delay research if research objectives can still be met at a later time without loss of scientific integrity.

#### Potential Participants (Individuals and Communities)

Disaster survivors, generally speaking, have experienced a difficult event and endured significant losses, of varying kinds and degrees. Scholars over the last four decades have documented the experiences of survivors including their painful displacements, economic losses, emotional distress, family separation, and personal losses. The COVID-19 pandemic has resulted in family separation as a result of physical distancing policies, and banned or restricted travel. Abrupt university closings left many college students, especially foreign students, with distress as many found themselves evicted from their dormitories leaving them stranded, and in some cases homeless, during the highest peak of the pandemic. Emotional trauma was heightened with the large number of deaths from COVID-19 with family members unable to visit their sick and dying loved ones or honor their lives with traditional funerals. Psychological impacts are a hallmark of disasters often with lasting emotional scars for survivors. In Hurricane Katrina, for example, Gulf Coast residents endured an abrupt displacement that scattered them across the USA, leaving them homeless and distraught, struggling to rebuild their lives amidst great uncertainty [13].

Special considerations for communities and individuals should not deter IRBs from approving disaster-related research but should serve as a source of reference for estimating actual risk and providing additional safeguards for potential participants. Certain characteristics have been identified to increase a participant’s potential for adverse outcomes in disaster research, including preexisting distress or mental illness, age, history of multiple trauma exposures, social vulnerability, and physical injury [5••]. Therefore, capacity assessment tools may be utilized, and the capacity of individuals may need to be monitored over time. Research proposals should include training for researchers in identifying these characteristics and mechanisms to refer participants to helpful services, such as therapy or counseling. In the conduct of the NIEHS GuLF Study, for instance, field staff were trained to identify urgent mental health issues and if necessary, participants were referred to nearby Federally Qualified Health Centers or acute care facilities. In addition, emergency services were dispatched, or participants were connected to suicide prevention hotlines in response to homicidal or suicidal ideation [22•].

Special populations require additional consideration for protection against coercion and undue influence, as outlined by the federal regulations for the protection of human subjects. In addition, disaster research may involve potential subjects who, because of social circumstances, may be more susceptible to coercion [29]. For example, voluntary participation should be stressed when enrolling individuals such as firefighters, police officers, and emergency service workers, who may feel it is their civic duty to participate in research [5••]. These individuals may be even more prone to coercion if their superiors endorse the study or if they believe participation will be beneficial to their job or position. Literature has also cited circumstances where participation was influenced by researchers offering disaster assistance or monetary compensation [30]. Having a perspective of the circumstances should guide IRBs in deciding when payments or other forms of compensation incentivize or unduly influence participants. Researchers may want to consider a suitable level of remuneration commensurate with research participant time and effort and pay special attention to avoiding undue inducement under extreme post-disaster circumstances.

Previous disaster literature has also identified “therapeutic misconception” as a potential problem with disaster research, that is when a participant, or researcher, confuses the purpose of clinical care and research [31]. While the purpose of research is to increase general knowledge, clinical care confers direct benefits to a participant. Though participating in disaster research may provide a participant with collateral benefits (e.g., increased knowledge of available services, empowerment, and emotional relief), most disaster research is not clinical in nature [5, 32]. Therapeutic misconception can have detrimental consequences for all parties involved, and IRBs should determine that the informed consent procedures minimize the likelihood of confusion.

Another facet of IRB review is assessing the cultural competency of the research team. Cultural norms and spiritual practices can vary widely among communities [33], and the success of research efforts may depend upon a proper understanding of the values and preferences of the community. There may also be a history of unethical research in certain communities that have rightly led to mistrust of researchers. For example, the ethical abuses associated with the US Public Health Service Tuskegee Study have had far-reaching effects on the willingness of African-Americans to participate in medical research studies, prevention programs, and government initiatives for decades after the scandal [34] and continuing to this day. In Arizona, the misuse of samples collected from the Havasupai tribe led to a similar loss of trust in research among Native Americans [35]. The impact of such negative experiences between researchers and racial/ethnic minorities has contributed to a broad underrepresentation of these populations in clinical research. The implications of this lack of participation in clinical research are significant and may be reflected in the current pandemic where ethnic/racial minorities have suffered disproportionate impact in morbidity and mortality [36]. Ideally, research teams should have an established track record of working with the affected community. If possible, the research team should include members of the community (ex. religious organizations, educational associations, clubs, Panhellenic groups, city and county organizations, etc.) on the research team to assure that recruitment practices and study procedures are consistent with the values of the targeted community. In addition, care should be taken to assure that all study materials are translated into language(s) anticipated in the cohort and are written at an appropriate reading level. Written study documents should be coupled with effective oral communication throughout the informed consent process.

The process of informed consent to participate in research “requires: a competent decision maker; adequate disclosure and comprehension of pertinent information; and a voluntary decision” [37]. In assessing the consent process in a disaster study, investigators and IRBs should consider whether the situation or the vulnerability of the research participants requires any additional considerations. As outlined in the Federal Policy for Protection of Human Subjects (“Common Rule”), “an investigator shall seek such consent only under circumstances that provide the prospective subject or representative sufficient opportunity to consider whether or not to participate...” Given the transient nature of individuals in the disaster setting, (e.g., mass sheltering, relocation, and standing in long lines for assistance), consideration needs to be given in the consent process for allowing sufficient time for participants to consider their options for participating in a study. Additionally, it needs to be made clear that participating in the study does not affect their receiving disaster aid. If participant vulnerability is a concern, researchers may want to consider re-consenting participants weeks to months after initial enrollment as an additional tool to ensure ongoing maintenance of a robust informed consent process and to remind participants of the voluntary nature of study participation. For example, following a disaster caused by a natural hazard, considerations may be needed for remote consent as individuals could be relocated and research can be conducted remotely. In the case of a pandemic such as COVID-19, additional considerations may be needed for the safety of the research teams (e.g., requirements of physical distancing) while maintaining the integrity of the process of consent. The regulations, under the revised Common Rule, do allow for some flexibility in obtaining and documenting consent (45 CFR 46.116), and the FDA cited acceptable changes to consent practices in clinical trials during the COVID-19 pandemic (https://www.fda.gov/media/136238/download). It is still important to ensure the participants, or their legally authorized representative, are able to provide consent and understand the difference between research and standard medical care. IRBs should allow for creativity in methods to achieve the goal of providing and documenting appropriate informed consent, for example, using technology such as email, teleconferencing, videoconferencing, electronic signatures, or taking pictures of the signed consent document.

Finally, the consent process should address additional concerns about subject privacy and confidentiality in the disaster field. Jacobsen and Landau [38] describe ethical considerations for research conducted during humanitarian disasters and complex emergencies, including protection of privacy and confidentiality. Their analysis included articles from the Journal of Refugee Studies and focused on the use of local researchers in the field and if translation was involved. While the use of local researchers and translators is believed to yield better results and improve validity, their analysis revealed that breaches in confidentiality were more likely, especially if the local researcher knew the participant. Jacobsen and Landau point out that many researchers do not adequately consider how research methods in disaster areas could compromise participant privacy and confidentiality. Protocol review should include careful scrutiny of proposed research methods, including data collection logistics and potential victimization if confidentiality is breached.

#### Return of Research Results

The dissemination of research results, when available, and ongoing communication with participants and the community in which they live is essential throughout the life cycle of the study. While this axiom is true for all research, it can be particularly important in disaster studies because the translation of research findings may be instrumental in overcoming hazard vulnerabilities and increasing the resiliency of the affected community, especially during the phases of preparedness and mitigation for future events. It is, therefore, crucial that the review board assures the proposed research has a written plan with a potential timeline for the return of results to key stakeholders which may include the participant, the community, local city and state officials, and first responders. A recent National Academies of Sciences report recommends that all research involving human specimens include a plan for the return of individual research results and empowers the IRB to play a key role in the review of such plans. [39] These return-of-results plans should be developed during the research design phase and incorporated in the study protocol for full transparency. The plan should capture whether there are any “alert” values that should be reported immediately to an individual and stakeholders. For transparency, during the consent process, study participants should be informed about the timing and type of results that will be distributed whether the results are at the individual level or as a summary of findings. In some cases, such as biorepository studies, results may not be immediately available, and this should be described within the return of results plan. The importance of such plans during the current pandemic cannot be overstated. Timely results from diagnostic and antibody testing, therapeutic interventions, and vaccine trials are critical and may provide real-time actionable information for the participant, health authorities, and federal, state, or local governments. In situations such as hurricanes, fires, and chemical exposures that rarely require therapeutic interventions, a summary of findings will benefit individuals and communities in understanding their exposures and potential effects. The review board should also examine if the format for the return of research results is clear and appropriate for the audience, whether it is intended for communities, municipal governments, schools, health care organizations, or individuals. Investigators should think of creative and multiple ways for presenting the results (e.g., forums, public meetings, webinars, radio interviews, newsletters, and social media) as most individuals obtain their information from diverse sources. It is imperative that long-term studies which follow participants for many years after a disaster keep participants aware of any new developments that may be valuable and of interest to them. Ongoing effective communication with participants has advantages to the research team, as well, since it reduces study cohort attrition. Consistent with the dynamic nature of disaster research studies, return-of-results plans should not be static and should incorporate feedback from IRBs and community engagement throughout the study timeline.

Studies that utilize a community-based participatory research (CBPR) approach may be particularly helpful for encouraging and informing the delivery of research results in disaster studies. The CBPR model is a collaborative research method that maximizes engagement of key stakeholders including prospective participants from study conception to dissemination. A key principle of CBPR is the dissemination of research findings and knowledge to all partners [40]. The engagement of the community at all phases of the research enables an ongoing collaborative feedback loop which allows the tailoring of research results to meet the needs of research participants. An example of this interaction is the NIEHS HEAL (Head-off Environmental Asthma in Louisiana) study which collected data from home environmental assessments after Hurricane Katrina [41]. These environmental assessment reports were then provided to study personnel, asthma counselors, and community health workers which allowed targeted interventions to reduce and potentially eliminate asthma triggers in the home [42].

## Summary

Few IRBs and investigators have experience in the development and review of disaster research protocols and are likely to reflexively resort to the standard approval criteria when presented with disaster research protocols. While the standard review criteria are effective for the review of most research, it does not take into consideration the unique circumstances that surround disasters, which could potentially put participants at risk of harm. The importance of the IRB in the review of disaster research cannot be overstated as there is often urgency associated with initiating the research which can lead to errors and oversights. Through proper use of preparedness training and tools, and collaborative interactions with investigators, IRBs can effectively conduct the ethical review of research while being responsive to the time sensitivity that is needed for implementation of disaster-related research.

The concept of facilitating the ethical review of disaster research protocols by improving IRB preparedness in advance of disasters has recently been discussed [43] but there remain gaps in the understanding of effective IRB preparedness strategies and their practical application. While resources and tools for IRB member training in key areas such as human subjects’ protection, research ethics, and responsible conduct of research are integral to standard curricula, there are limited disaster preparedness training and resources available to IRBs. The Disaster Research Critical IRB Review Factors Model is a new approach to the review of disaster-related research that emphasizes contextual factors and considerations to strengthen ethical research practices.
